# Team Resilience Training in the Workplace: E-Learning Adaptation, Measurement Model, and Two Pilot Studies

**DOI:** 10.2196/mental.8955

**Published:** 2018-05-02

**Authors:** Joel B Bennett, Michael Neeper, Brittany D Linde, Gale M Lucas, Lindsay Simone

**Affiliations:** ^1^ Organizational Wellness & Learning Systems Fort Worth, TX United States; ^2^ ACEC Life/Health Trust Frisco, TX United States

**Keywords:** workplace, resilience, stress, quasi-experimental, experimental design, online learning, early intervention, questionnaire design, incentives, social support, psychological theory, gender

## Abstract

**Background:**

The majority of resilience interventions focus on the individual. Workplace resilience is a growing field of research. Given the ever-increasing interconnectedness in businesses, teamwork is a guarantee. There is also growing recognition that resilience functions at the team level.

**Objective:**

The objective of our work was to address three shortcomings in the study of workplace resilience interventions: lack of interventions focusing on group-level or team resilience, the need for brief interventions, and the need for more theoretical precision in intervention studies.

**Methods:**

The authors took an established evidence-based program (Team Resilience) and modified it based on these needs. A working model for brief intervention evaluation distinguishes outcomes that are proximal (perceptions that the program improved resilience) and distal (dispositional resilience). A total of 7 hypotheses tested the model and program efficacy.

**Results:**

Two samples (n=118 and n=181) of engineering firms received the Web-based training and provided immediate reactions in a posttest-only design. The second sample also included a control condition (n=201). The findings support the model and program efficacy. For example, workplace resilience was greater in the intervention group than in the control group. Other findings suggest social dissemination effects, equal outcomes for employees at different stress levels, and greater benefit for females.

**Conclusions:**

This preliminary research provides evidence for the capabilities of e-learning modules to effectively promote workplace resilience and a working model of team resilience.

## Introduction

### Web-Based Resilience Training

Recent national studies indicate increases in worker stress [1,2] and its impact on disease [3] and health and productivity costs [4]. The increase in stress corresponds to growing interest in the topic of resilience within business, popular culture, and public health. Self-help books have titles such as The Bounce Back Book [5-7]. Trainings designed for enhancing military resilience [8,9] include mobile apps [10]. Business training continues to grow [11-13] as do strategies promoting urban and institutional resilience [14]. Although resilience operates across individual, workplace, and social levels [15,16], most studies assess resilience as an individual trait [17,18].

This individual-level focus ignores research showing psychosocial factors impact stress and health [2,4]. Effective well-being solutions often target social and systemic factors [19-21], yet resilience interventions focus on individuals (see meta-analysis [22]). Recent reviews of workplace resilience (WR) interventions—a meta-analysis of 37 studies [23] and a systematic review of 14 studies [24]—concluded that methodological weaknesses, lack of conceptual clarity, and measurement inconsistency limit efficacy of these interventions. Although training effects are small, resilience building via computer-based formats was seen to have potential. The studies described in this paper sought to promote a more proactive approach to resilience in the workplace [25]. WR can be defined as the overall ability of employees to “bounce back” from an obstacle or negative event in the workplace (eg, missing a deadline, workers out sick) and, together, use various resources to address that obstacle in a positive manner (eg, time and project management, wellness, and employee assistance services).

The study of WR interventions may be advanced in several ways. First, interventions could more fully address resilience at the group or team level. Indeed, recent studies support resilience as a team phenomenon [26-30]. Second, most interventions are classroom-based and lengthy, ranging from 2.5 days to 5 to 11 weeks [24]. Although Web-based learning (ie, e-learning) makes training less costly and easier to access, there is a need to adapt classroom programs—especially those that are evidence-based—into e-learning format. Scientific knowledge about effective e-learning offers guidelines for these adaptations [31,32]. Third, intervention models require more theoretical precision [22,24]. Definitions of resilience lack agreement, [33,34] likely because resilience is itself a multilevel construct [35,36] and comprises many resources (eg, social support, skill confidence, and stress management) [9,16]. Although these resources can be found both within persons (ie, an internal trait) [37] and among peers in the work environment [38], previous intervention models fail to distinguish these 2 basic levels.

### Team Resilience

To address these needs, we selected Team Resilience (TR), cited previously and also independently recognized as evidence-based by the national government [39], for adaptation to e-learning. TR is a valuable construct in the modern workplace; given our ever-increasing interconnectedness within businesses, teamwork is almost a guarantee. The original TR study was a randomized longitudinal clinical trial, based on theory [40,41]. Findings suggest promise for replication, including reductions in stress, substance use, and social-level problems among a high-risk sample of restaurant workers [40,42,43]. Personal resilience increased across training sessions [40]. TR also improved teamwork, helping workers to be more compassionate toward others [41]. Most workers who reported these positive gains had previously indicated having work-related alcohol problems. Similarly, in Vanhove et al’s review [23], resilience interventions that were designed to target high-risk samples may be more likely to show longer-term gains. Indeed, compared with control respondents, TR participants showed significant decreases in heavy drinking at 12 months [42].

TR promotes social dissemination, or peer-to-peer sharing, of resilience. Employees assessed at follow-up who were not exposed to the original training or employed at the time of the intervention had reduced stress and exposure to counterproductive work behaviors (CWBs) [43]. CWBs included theft, bullying, physical fights, coworker rudeness, and arguing. Employees who did not attend training also reported benefits via coworkers, as a type of “spill-over” or “ripple” effect [44]. Specifically, 24% of respondents at 12 months who had heard about TR (but never themselves participated) reported personal benefit; 31% of respondents reported seeing their coworkers benefit. The clinical trial occurred during the 2008-2009 economic downturn, with restaurant closings nationwide, including several in the sample. The reductions in stress—especially during adversity—speak to the resilience function of TR.

### Team Resilience: A Promising Training Framework for Adaptation

Several factors suggest that TR is promising for e-learning: a detailed and modular training manual; evidence for effectiveness; potential for social dissemination; advances in our understanding of team or social resilience; and a strong theoretical basis. A core feature of TR was the inclusion of participant exercises on 5 resilience competencies [40,41]. This “Five C” framework was developed from studies, suggesting resilience exists *both* in the individual and among their social resources [45-52]. The following are the Five Cs:

- Centering (positive coping skills)
- Confidence (self-efficacy and positive thinking)
- Commitment (mental toughness, perseverance, and value-based behavior)
- Community (social support, connectedness, and unit cohesion)
- Compassion (empathy, perspective taking, and nurturing).

In their meta-analysis, Leppin et al [22] stated that the Five C framework is supported theoretically. Other studies validate the Five C’s competencies. For example, the commitment aspect led to more positive attitudes under conditions of adversity [53]. Moreover, a resilience training for Army personnel addressed compassion and community aspects through improved empathy and reduced loneliness [54].

To modify the previous classroom training for e-learning delivery, we reviewed original TR training manuals and studies on effective e-learning features. Such features include customization to the user or workplace, interactive elements, regular quizzing, personalized feedback, multimodule information, and psychoeducational resources [31,32,55]. The TR manuals were originally created by the first author, allowing for a more informed and encompassing transition to the Web-based presentation.

### A Guiding Model for Measurement

As noted, progress in the study of resilience has been limited because of lack of conceptual clarity. Recently, the use of “wise interventions” [56] suggests designing programs that are psychologically precise, brief, and aim to alter self-reinforcing processes that unfold over time. We borrow from this “wise intervention” approach to propose a limited model to study only the short-term effects of the intervention. By focusing narrowly, we hope to discern how a brief electronic training might work.

**Figure 1:**
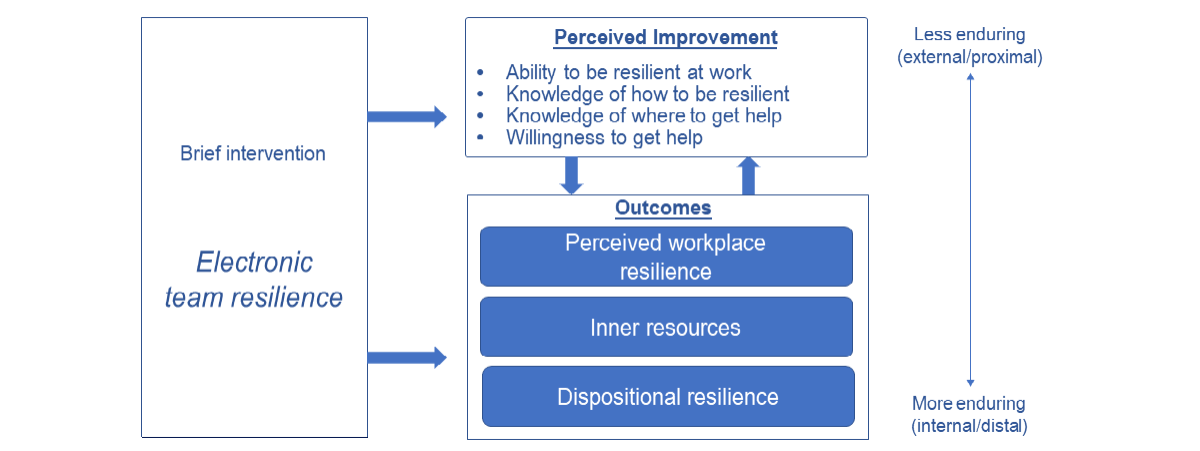
Working model for brief intervention evaluation.

Figure 1 shows our working model, which only targets immediate psychological changes. As is typical in workplace training evaluations, participants are expected to report improvement in content areas targeted by the intervention, namely, their own resilience. We focus on 4 areas of *perceived improvement* (PI): ability to be resilient at work, knowledge of resilience, of where to get help, and willingness to get help. As Figure 1 notes (top), such PI implies a relatively immediate, proximal, and external response to training.

These perceived changes should correlate with proximal outcomes, yet ultimately affect distal outcomes also targeted by the intervention. We propose a “situational-to-dispositional” ordering of outcomes, from *perceived WR* (this is an employee’s ability to adapt to workplace stress and associated perceptions that their workplace and coworkers contribute to their resilience) to *recognition of inner resources* (IR) for resilience and to an enduring trait or *dispositional resilience* (DR). An employee’s recognition of her own WR should affect her behavior in a positive way, following Ajzen’s theory of planned behavior [57].

First, as a result of the training, participants should perceive that their workplace—including their coworkers—provides them with resources for resilience (ie, WR). For example, TR makes multiple references to their Employee Assistance Program (EAP) and other workplace health resources. Although newcomer employees learn about these resources during orientation, they may be overwhelmed with information. Reminders (in the context of resilience) are designed to heighten awareness of their workplace as a resource.

Second, the adapted TR guides users to focus on *internal resources* (eg, confidence, commitment) through self-reflection exercises, a common feature of other resilience programs (eg, [21,22,54]). Participants are expected to perceive that such resources help them deal with challenges. Relatively speaking, these IR are more internal and enduring than perceived changes in the work environment. However, they are less enduring than the more endogenous trait of resilience or DR. We do not expect a brief intervention, such as that described in this paper, to change DR in an immediate context. However, the proposed model suggests that short-term changes (ie, improvement attributed to the training) may bolster longer-term increases in internal resilience. Hence, DR is a third factor in our model.

Furthermore, resilience may best be measured in the context of adversity (eg, *adaptive cycle*) [14]. Thus, resilience interventions may be more effective in populations with high stress [23]. Accordingly, a valid test of a resilience program should occur in the context of exposure to adversity. Ideally, such a test would first randomly assign 2 groups: an intervention group and a no-intervention control group. Both groups would then be exposed to a work-related challenge or adversity, with the intervention group then receiving the training. Hypothesized outcomes would be: (1) the intervention group would cope better with the challenge and (2) such coping would be best predicted by the most proximal variables in the model (eg, PI, followed by perceived WR). Before conducting such a randomized trial, the proposed model can yield insights through an initial feasibility assessment or quasi-experimental pilot test.

### Goals and Hypotheses

The primary goals of this pilot project were to assess (1) the feasibility of condensing a classroom training into e-learning and (2) employees’ reactions to the program using the working model described previously (see Figure 1).

A total of 7 hypotheses were derived from these goals:

H1: Employees receiving the pilot program would report improvements beyond those that might be expected by chance (reporting improvements beyond “none” or “little” on a 5-point scale; ie, greater than mean 2.5).

H2: Compared with a nonrandomized control group, employees receiving the pilot program would self-report greater levels of improvement in resilience.

As a study of self-reported resilience, we explore a new measure to distinguish ratings of WR, IR, and DR. H3 and H4 reflect these distinct outcomes:

H3: Compared with a control group, employees exposed to e-learning will self-report greater levels of resilience, especially for WR.

H4: Because the intervention focuses on workplace and TR, it is hypothesized that WR will most strongly correlate with PI. We also expect that (1) reported improvements would correlate positively with WR, while holding the other 2 forms of resilience constant; and (2) these relationships will only be present in the program group.

To support construct validity, we examined other distinctions between workplace and DR. Accordingly, variables should relate to those variables one would expect them to relate to (ie, convergent validity) and not relate to those expected to be different (ie, discriminant validity). For convergent validity, measures of resilience would be expected to show an inverse relationship with recent stress. For example, employees who have either DR or WR might be less inclined to experience recent stress; therefore, we examined how both of these correlate with stress:

H5: Recent stress will be inversely correlated with DR and WR.

This study purposely assessed only short-term reactions to training. As noted previously, research suggests resilience training may be more effective in a high-stress sample [23]. Thus, we did not expect short-term differences between workers recently experiencing high versus low stress. As a brief primary prevention approach, the program was not designed to have large enough “dose effects” to address significant stress. Accordingly:

H6: Self-reported PIs and resilience would not be different between employees with high and low stress.

One aspect of the original TR is its potential for social dissemination [43]. In this study, employees from different firms participated in 2 sample time frames (2015 and 2017). We tracked whether those employees in the 2017 sample came from firms that had previously used the program. Social dissemination suggests that employees from firms with previous use would gain more from the program. Specifically:

H7: Compared with employees from firms with no previous exposure to the resilience training (ie, no employees from the firms in 2017 completed the training in 2015), employees from firms that had exposure will show greater outcomes (greater PI, greater resilience).

This paper also explores other factors related to the sample. First, we examine whether the training is more effective for women. A recent meta-analysis suggested that women may benefit more from increases in resilience [18]. Second, the original TR was also developed for young, at-risk restaurant workers. We test the current adaptation in an adult workplace population that differs from the original sample. The same information presented in the original TR module is included in this project. However, we created a module that benefits from the advantages that e-learning content provides (more details below). Hence, this study aims to test the generalizability of both content of the program in question and of the target population for this research.

## Methods

### Sample

Participants were recruited in 2015 and 2017 from firms within a national engineering association. Most firms were using a wellness benefit through the association for between 1 and 4 years. All 2015 participants were recruited to receive the program. In 2017, participants were also recruited to receive the program; a month later, only those who had not previously participated were then eligible to participate in a control sample. Table 1 summarizes demographics of the samples. Although some firms in the 2017 sample had previously participated in 2015, no data from those who had previously completed this training were used in analyses (ie, 17 participants were removed from the 2017 sample to prevent any data contamination).

#### Sample 1 (2015)

A total of 217 participants from 40 firms began the survey; 174 ultimately completed it. Firm size ranged from 6 to 142 participants. The average number of participants from each firm was 4.28 (SD 3.91). The sample was 56.9% (99/174) female, and the modal (40%) age group was 26 to 40 years. Most participants had at least a Bachelor’s degree (78.7%; 117/174).

#### Sample 2 (2017)

A total of 121 experimental participants began the survey, and 118 ultimately completed it. The control group consisted of 186 individuals. More men (64.5%; 120/186) than women (35.5%; 66/186) participated in the control group (χ^2^_2_=19.0, *P*<.01). Overall, most participants had at least a Bachelor’s degree (74.0%; 225/304). Moreover, 32 firms, ranging in size from 13 to 67 employees, participated in the intervention (mean per firm 3.69; SD 3.98). A total of 31 firms, ranging in size from 6 to 234 employees, comprised the control group (mean per firm 6.13; SD 7.33). The control sample was acquired after completion of the intervention. Specifically, the recruitment invitation asked employees to participate only if they had not previously done so.

### Procedures

Program participants were recruited by an email sent from the local “Wellness Champion” within their firm. A “Wellness Champion” is an employee within the business that takes it upon themselves, whether formally or informally, to help coworkers take advantage of the company’s wellness resources. These champions also encourage participation in wellness programs, not unlike the one described in this paper. The association’s wellness director first sent an email template to each champion, who then distributed the email to employees. The email invited confidential participation in a Web-based, e-learning resilience module and a postsurvey questionnaire.

On receipt of the invitation email, participants clicked a URL link and entered their name and email address (used only for tracking and incentive purposes) to begin the training program. Participants had access to the modules at all times, via desktop, mobile, or tablet. More details about the program are discussed below.

**Table 1:** Demographic breakdown of participants in all samples.

Demographics		Sample 1 (2015; n=174)	Sample 2 (2017; n=304)	
		Program only	Program (n=118)	Control (n=186)
**Gender n (%)**				
	Male	75 (43.1)	54 (45.8)	120 (64.5)
**Age in years, n (%)**				
	18-25	18 (10.3)	13 (11.0)	29 (15.6)
	26-40	70 (40.2)	58 (49.2)	75 (40.3)
	41-50	35 (20.1)	22 (18.6)	38 (20.4)
	≥51	51 (29.3)	25 (21.2)	44 (23.7)
**Education n (%)**				
	Less than high school	1 (0.6)	7 (5.9)	3 (1.6)
	High school	4 (2.3)	4 (3.4)	10 (5.4)
	Some college	32 (18.4)	17 (14.4)	38 (20.4)
	Completed college	98 (56.3)	56 (47.5)	93 (50.0)
	Advanced degree	19 (22.4)	34 (28.8)	42 (22.5)

#### Incentives—Samples 1 and 2

The same program was administered in 2015 and 2017. However, the 2 samples received different program completion incentives. Sample 1 received both individual- and firm-level incentives. Participants were informed at the beginning of the survey that they would be entered into a raffle to win a fitness armband. Sample 1 firms with the highest proportion of participants earned a US $200 award to use their wellness program. Sample 2 participants received US $5 for completing the program and an additional US $5 for completing the survey. Sample 2 control group participants received a US $5 Amazon.com gift card for filling out the survey after reading a short article on resilience tips.

#### Firm Participation Over Time

For purposes of estimating social dissemination, we assessed how many 2017 participants came from 2015 firms. Of the 118 employees participating in the 2017 program, 36 participants came from 16 firms with no prior participation, 10 from 5 firms with one prior participant, and 54 from 11 firms with 2 or more participants. Of 201 employees participating in the 2017 control condition, corresponding numbers were 95 participants from 17 firms with no prior participation, 28 from 4 firms with one prior participant, and 56 from 10 firms with 2 or more participants. In 2017, 20 firms participated in the program condition, 19 firms participated in the control condition, and 12 firms participated in both conditions.

#### Web-Based Team Resilience

The e-learning module sought to increase participants’ ability to be resilient in the workplace, their knowledge of resiliency, their awareness of resources, and their willingness to access those resources when necessary. The program consisted of tips and strategies around building “5 Cs of Resilience” (ie, Centering, Commitment, Community, Compassion, and Confidence).

We followed 6 design goals for developing the electronic module: (1) *brevity, ease of access, self-paced*—limit to 45 min, viewable in segments, with user ability to leave and return anytime; (2) *mimic team environment*—show scenarios of characters who are part of a team, with exercises guiding users to reflect on their own coworkers; (3) *tailored feedback*—require users to complete a self-assessment and receive feedback on personal resilience; (4) *customized EAP resources*—give access to behavioral health resources (EAP); (5) *guided facilitation*—provide a facilitator/narrator who shares personal stories around resilience; and (6) *team assessment of strengths*—require users to reflect on the Five Cs in their coworkers and also their perceptions of how coworkers view the user’s own strengths.

**Figure 2:**
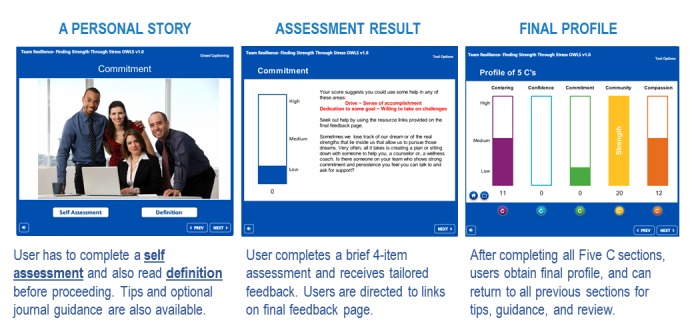
Core sequence of each Five C component and final profile.

The module was created using Articulate Storyline (Articulate, New York, NY) and included video, audio, interactive exercises, and quizzes in 4 sections: (1) *Welcome* with videos introducing characters explaining the importance of a healthy team; (2) *Best Coworker Exercise* where users review the Five Cs as qualities that exist in coworkers; (3) *Resilience* where users (a) watch 2- to 3-min video vignettes of employees who used one of the Five Cs to overcome a health, stress, or performance challenge (often with the help of a coworker); (b) complete a self-assessment on the Five Cs; (c) review definitions, tips, and journal exercise reflecting on the Cs; and (4) a *Summary* where users (a) see a final profile of their previous self-assessments and (b) rate their team on the Five Cs. Figure 2 shows the core sequence of each Five C component.

Participants in both samples were given 4 to 6 weeks to complete the program and accessibility from any computer or mobile device. The program contained 55 slides; the minimum number of slides to be viewed to receive credit for the program was 38, but most participants viewed at least 45 slides. Immediately after program completion, participants were automatically provided the survey link.

#### Control Condition

On completion of the 2017 program condition, wellness champions sent an email to employees asking for input from those who had never completed any wellness program. Participants filled out a survey after reading a one-page article featuring 8 tips on how to “build resilience” (eg, sleep, relaxation, ask for help). Participants were given 1 week to complete the survey. Any individual who had previously participated was removed from data analyses.

### Measures

A survey assessed participants in 4 areas: (1) demographic information; (2) PI or impact of the resilience module (eg, “My willingness to: be more resilient at work, use resilience resources, has…”); (3) measures in 3 areas of resilience: WR, IR, and DR; and (4) one satisfaction item (“Overall, how satisfied were you with the online module?”). Response options were on 5-point Likert scales for PI (1—“Stayed the same” to 5—“Improved greatly”) and for resilience (1—“Not true about me” to 5—“Very true about me”). WR was evaluated using 3 items derived from a review of recent writings on the topic [22,23,30] and developed specifically to assess TR training objectives. Items asked whether the workplace/coworkers contributed to one’s own resilience and to knowing workplace resources to help address hardship. In addition, 2 IR items were developed to further examine construct validity, ie, to distinguish between external resilience in the work setting and inner resilience. Items align with resilience concepts that informed the Five C model [45-52] and related to the self-assessment exercises (eg, “I have the inner resources to deal with life’s challenges”). DR was evaluated using 4 items adapted from the Connor-Davidson Resilience Scale [37] that had high loadings in their original study (eg, “I can deal with whatever difficulties come my way.”).

Reliabilities, assessed by combining data across all samples, were PI: alpha=.92; WR: alpha=.68; IR: alpha=.59; and DR: alpha=.86. The 2017 survey included an item asking about stress: “How much has stress hurt your ability to stay healthy and productive in the past month?” Response options ranged from “not at all” (1) to a “great amount” (5). This survey was given to participants on completion of the training program.

## Results

We compared responses for all items for samples 1 and 2 and found no significant differences (all *P*values>.05). Hence, samples 1 and 2 were combined into a single program group (n=299).

### Perceived Improvement

H1 proposes the idea that the mean PI score would be greater than chance. A mean rating of 2.5 was used as a conservative baseline, as it represented the mid-point between improving “slightly” and “some.” For the program group, ratings on all 4 items reflected significant improvements (ability—*t*_298_=7.49, *P*<.01; knowledge of how to be more resilient—*t*_298_=12.82, *P*<.01; knowledge of where to get help— *t*_298_=10.83, *P*<.01; willingness to use the resources— *t*_298_=9.22, *P*<.01.). For the control group, only one item differed from 2.5, *t*_200_=2.26, *P*=.03. This item—knowledge of how to be more resilient—was also highest rated, suggesting that both e-learning and the reading improved knowledge of resilience. In support of H2, ratings on all 4 items reflected higher ratings on improvements for program (overall mean 3.08; SD 0.88) than control (mean 2.54; SD 1.06), *t*_498_=6.19, *P*<.001.

### Resilience

H3 predicted that, across all 4 measures, resilience would be greater for program versus control participants. As expected, the strongest effect was for WR; program (mean 3.85; SD 0.68) versus control (mean 3.11; SD 0.75), *t*_498_=11.44; *P*<.001.

H4 proposes that WR would correlate most strongly with PI. Again, training objectives sought to improve both TR *among* coworkers and access to resources *within* the work setting. As shown in Table 2 (left half), compared with IR and DR, WR had the only consistent relationship with PI. These relationships held across both program and control conditions and also when controlling for the 2 other aspects of resilience. There was an unexpected negative partial correlation between DR and PI (*r*_partial_=−.19), but other findings were consistent with H4.

H5 predicted that DR would have the strongest relationship with stress (measured in sample 2). Table 2 (right half) shows relationships between the resilience measures and recent stress. H5 is confirmed for the program group (correlation between DR and stress, *r*=−.56). This relationship is maintained (*r*_partial_=−.46) after controlling for the other aspects of resilience. The relationship between both WR and IR and stress become insignificant after controlling for DR. In contrast to confirming H5 in the program group, there were no significant correlations between any resilience measures including DR and stress within the control group.

### Recent Stress (Before the Program)

There was no difference in stress levels between program (mean 2.58; SD 0.96) and control (mean 2.60; SD 1.04) participants. Because the brief resilience program focused on helping employees with stress, we explored whether those across 3 levels of stress (low, medium, and high) benefitted from the program (Table 3). Participants with low stress indicated that they felt stressed in the last month either “not at all” or “a little”; medium stress answered “some” or “much”; and high stress answered “a great amount.”

**Table 2:** Relationship of resilience measures to perceived improvement and stress.

Outcome	Perceived improvement				Stress^a^			
	Program		Control		Program		Control	
	*R*	Partial *r*	*r*	Partial *r*	*r*	Partial *r*	*r*	Partial *r*
Workplace resilience	.24^b^	.24^b^	.47^b^	.44^b^	−.36^b^	−.13	−.08	−.05
Inner resources	.09	−.02	.27^b^	.18^c^	−.28^b^	−.02	−.05	.01
Dispositional resilience	.06	−.06	.14	−.19^b^	−.56^b^	−.46^b^	−.07	−.04

^a^Only measured in sample 2.

^b^*P*<.01.

^c^*P*<.05.

**Table 3:** Comparing program and control outcomes at different stress levels (sample 2). Adjusted means are shown, controlling for gender, age, and education.

Analysis		Stress level^a^						Analysis of variance		
		Program (n=118)			Control (n=186)			Main Effect		Interact
		Low	Med	High	Low	Med	High	Stress	Program	
Subsample, n		54	48	16	90	62	34	–	–	–
**Outcome**										
	Perceived improvement	2.99	3.16	3.08	2.39	2.83	2.35	NS^b^	15.66^c^	NS
	Workplace resilience	3.91	3.76	3.35	3.19	3.08	2.98	5.17^c^	42.37^d^	NS
	Inner resources	4.09	3.89	3.59	3.83	3.73	3.62	5.49^c^	NS	NS
	Dispositional resilience	4.31	4.20	3.31	4.08	3.97	3.88	18.33^c^	NS	10.94^c^
	Satisfaction	3.41	3.60	3.44	3.12	3.19	3.00	NS	6.27^d^	NS

^a^Low: not at all or a little; med: some; high: much or great amount.

^b^NS: nonsignificant.

^c^*P*<.01.

^d^*P*<.05.

**Table 4:** Gender differences in outcomes.

Item	Program, mean (SD)			Control, mean (SD)		
	Female (n=149)	Male (n=126)	*t* value (*df*)	Female (n=66)	Male (n=120)	*t* value (*df*)
Perceived improvement	3.10 (0.90)	3.08 (0.82)	0.19 (273)	2.66 (1.08)	2.46 (1)	0.63 (184)
Workplace resilience	3.96 (0.59)	3.81 (0.64)	2.02^a^ (273)	3.11 (0.72)	3.12 (0.66)	0.10 (184)
Inner resources	4.07 (0.60)	4.08 (0.58)	0.14 (273)	3.79 (0.61)	3.74 (0.70)	0.49 (184)
Dispositional resilience	4.19 (0.60)	4.27 (0.53)	1.16 (273)	3.88 (0.57)	4.07 (0.55)	2.23^a^ (184)
Satisfaction	3.67 (0.87)	3.44 (0.88)	2.17^a^ (273)	3.30 (1.04)	3.02 (0.97)	1.84 (184)

^a^*P*<.05, both samples.

A two (condition) by three (stress level) analysis of variance (ANOVA) was conducted while controlling for demographics (gender, age, and education). There was a main program effect for PI, WR, and program satisfaction. Consistent with correlations in Table 2, all 3 types of resilience differed across stress levels. Program satisfaction was equal across stress groups. H6 proposed the program would be equally effective across stress levels. Hence, a test of the interaction term in the ANOVA should show no significance. Table 4 supports H6 with one exception for DR. Although the overall pattern shows decreasing DR as stress levels increases, significantly lower DR ratings occurred for high-stress employees in the program condition (mean 3.31). This may be because of the small sample size (n=16) in this cell (see Table 3 for other findings).

### Assessing Dissemination Effects

Employees in the 2017 sample worked in firms that had different amounts of previous exposure to the resilience training. Both the 2015 and 2017 datasets allowed comparison of employees from “nonexposed” firms to those firms where employees may have learned something from previous participants. H7 posited that PI and WR would be strongest among employees from firms with two or more previous participants versus only one or no previous participants.

No differences in PI or WR were found across these 3 levels of previous exposure for either the program or control groups. However, stress was lowest among program participants coming from firms where two or more employees had previously had the training: none (mean 2.78; SD 0.96), one (mean 2.7; SD 1.06), or more than one (mean 2.39; SD 0.86), *F*_2,81_=3.96, *P*=.02. In addition, employees from firms with no previous exposure were the most satisfied; none (mean 3.89; SD 0.82), one (mean 3.3; SD 0.82), or more than one (mean 3.31; SD 0.93), *F*_2,97_=4.93, *P*<.01. Comparisons within control group found no differences in stress (*F*_2,176_=1.32, *ns*) or satisfaction (*F*_2,176_=1.97, ns).

### Gender Differences

Additional tests, shown in Table 4, explored differences between women and men. In the program group, females scored higher than males in both WR and satisfaction. However, in the control group, males scored higher than females in DR.

## Discussion

A total of 7 hypotheses were tested through an exploratory pilot study. Results generally support the conclusion that a brief Web-based resilience program can lead to proximal improvements in resilience as a social resource within work settings. This finding is supported by tests of H1, H2, and H3; the latter hypothesis included a control group comparison. Experimental versus control study comparisons showed positive outcomes for PI, WR, and satisfaction.

Stress has an impact on employee health and performance, and employees have a need for effective programs to address stress. Ideally, employers who purchase or promote such programs should know that their investments are wise ones, based on evidence [57]. Fortunately, there has been recent growth in the science of Web-based interventions to improve employee well-being and mental health (“digital mental health”) [58,59]. Previous reviews point to several characteristics that should make a WR program most useful: a basis in a theoretically precise model; relative brevity; ease of access; personalized feedback that engages users; some use of points or incentives for participation; and previous evidence for clinical effectiveness.

We add this to list the need for programs that enhance social well-being and educate workers about the impact of their own health on coworkers. This social focus can enhance potential dissemination or ripple effects [19], further adding to the efficacy of employer investment. This study incorporated all these characteristics into an e-learning design. We detailed the development and content of a new intervention based on an established evidence-based program. Our study tested the effectiveness of this new intervention, albeit without the inclusion of a fully randomized clinical trial and with limitations as discussed below.

In support of H4, PI (ratings of how much the training improved resilience at work) showed a correlation with WR. Several factors could account for these results. In particular, TR was designed to be distributed to employees *within* their workplace. It follows that the training would influence participants’ responses toward items that evaluate WR, but not as much with items that assess DR or IR.

In support of construct validity, only DR was significantly and inversely correlated with recent stress, after controlling for the other resilience measures (Table 2, H5). H6 proposed that, because of the short-term focus of the training, the training was not expected to be differentially effective for those with low versus high stress. Additional tests support this hypothesis. However, those with low or moderate stress may benefit from a brief intervention (see Table 3).

Overall, findings also support a newly proposed working model for brief intervention evaluation that distinguishes proximal outcomes from longer-term dispositional resilience. Given the lack of clarity in previous resilience intervention studies, we hope that the findings from this study lead future researchers to clearly distinguish WR from dispositional measures.

Of special interest was a test of dissemination effects. We compared sample 2 (2017) employees from firms with no previous (2015) exposure to the resilience training with employees from firms that had previous exposure. H7 claimed that the previously exposed group would show more positive outcomes. Results did not show any differences among the variables in the working model for brief intervention evaluation (eg, PI, WR). However, those in the program group who came from firms with previous exposure reported less recent stress and lesser program satisfaction. It is difficult to say whether these employees benefited from their previous coworker’s experience or whether those who were less stressed also self-selected into the program. However, the same finding was not apparent in the control sample, suggesting that TR may have made previous coworker’s exposure more salient to current and new participants.

This study contained several important limitations. First, we used a nonrandomized quasi-experimental design, with a self-selected, convenience sample. Differences found between conditions may be due to preexisting characteristics in these groups or some other sampling artifact. This includes types of incentives used (between experimental and control groups), varying study time frames, and a sole focus on engineering firms. Although the findings of intervention-control comparisons are strengthened by the fact that there were 2 samples in the intervention condition, the control group was highly selected. Employees were recruited who specifically had not participated in any previous wellness program and who were asked to participate partly to share why they had not previously engaged in previous programs.

Other limitations may be considered in light of the pilot nature of the study. The measures that were used were themselves piloted, without full-scale development and factor analyses. Although the DR measure was adapted from Connor and Davidson [37], it was deliberately shortened. Additional items should be used to improve all scale reliabilities, especially more comprehensive and validated measures of recent stress or exposure to adversity.

Another limitation of this study was its focus on only immediate or proximal reactions. A more useful test would assess longer-term outcomes, especially taking ongoing stress into consideration. However, the workplace training literature suggests that utility types of reactions—as used here with PI—may correlate with more distal outcomes (eg, program satisfaction) [60,61]. The PI and the WR measures focused on knowledge, ability, and willingness to use WR, and results showed that the intervention improved the perceived utility of such resources. The improved perception of the workplace is noteworthy as the actual impact of training may depend on how much employees feel their organization cares about them [62].

Finally, the organizations sampled in this study varied in size, and none of them employed a substantial amount of people, compared with large organizations (ie, 500+ employees). It could be safely assumed that there is a positive linear relationship between the number of employees and the number of teams that are within the organization, which could result in stronger findings (eg, more dissemination throughout employees). This would be an interesting avenue to explore in further research, especially as research on small business wellness is limited [63].

Overall, this study is best viewed as an exploratory pilot. However, it makes several contributions. First this work also included both a new theoretical model and an e-learning extension or adaptation of an established classroom training. Furthermore, besides adapting the classroom training into an e-learning training, this work also tested the intervention on a different occupational sample (engineers) than the original study (restaurant workers). Findings suggest further and more rigorous tests of the model may be promising for the science of Web-based workplace TR training.

## Abbreviations

ANOVA: analysis of variance
CWB: counterproductive work behavior
DR: dispositional resilience
EAP: Employee Assistance Program
IR: inner resources
PI: perceived improvement
TR: team resilience
WR: workplace resilience
